# Risk factors for severe bleeding events during warfarin treatment: the influence of sex, age, comorbidity and co-medication

**DOI:** 10.1007/s00228-020-02856-6

**Published:** 2020-03-28

**Authors:** Diana M. Rydberg, Marie Linder, Rickard E. Malmström, Morten Andersen

**Affiliations:** 1 Department of Medicine Solna, Karolinska Institutet, Stockholm, Sweden; 2 Clinical Pharmacology, Drug Evaluation Unit, L2:04, Karolinska University Hospital Solna, 171 76 Stockholm, Sweden; 3 Centre for Pharmacoepidemiology, Department of Medicine Solna, Karolinska Institutet, Stockholm, Sweden; 4 Department of Drug Design and Pharmacology, Faculty of Health and Medical Sciences, University of Copenhagen, Copenhagen, Denmark

**Keywords:** Anticoagulants, Warfarin, Women, Men, Adverse drug events, Severe bleeding, Haemorrhage, Sex differences

## Abstract

**Purpose:**

To investigate risk factors for severe bleeding during warfarin treatment, including the influence of sex, age, comorbidity and co-medication on bleeding risk.

**Methods:**

Patients initiating warfarin treatment between 2007 and 2011 were identified in the nationwide Swedish Prescribed Drug Register, and diagnoses of severe bleeding were retrieved from the National Patient Register. Hazard ratios (HR) with 95% confidence intervals (CI) for severe bleeding were estimated using multiple Cox regression adjusting for indications and including covariates age, sex, comorbidities and co-medications. Interactions between sex and other covariates were investigated.

**Results:**

The study cohort included 232,624 patients ≥ 18 years (101,011 women and 131,613 men). The incidence rate of severe bleeding was 37 per 1000 person-years, lower among women than men with an adjusted HR (95% CI) of 0.84 (0.80–0.88). Incidence of bleeding increased with age, HR 2.88 (2.37–3.50) comparing age ≥ 80 to < 40 years, and comorbidities associated with the highest risk of severe bleeding were prior bleeding, HR 1.85 (1.74–1.97); renal failure, HR 1.82 (1.66–2.00); and alcohol dependency diagnosis, HR 1.79 (1.57–2.05). Other comorbidities significantly associated with bleeding events were hypertension, diabetes, peripheral vascular disease, congestive heart failure, liver failure, stroke/TIA, COPD and cancer.

**Conclusion:**

Most of the well-established risk factors were found to be significantly associated with bleeding events in our study. We additionally found that women had a lower incidence of bleeding. Potential biases are selection effects, residual confounding and unmeasured frailty.

**Electronic supplementary material:**

The online version of this article (10.1007/s00228-020-02856-6) contains supplementary material, which is available to authorized users.

## Introduction

There are several known risk factors for bleeding during treatment with oral anticoagulants, such as age, chronic comorbidities, prior bleeding and certain co-medications which are included in the HAS-BLED score [1]. Sex is not included in this risk score, and conflicting results have been found in different populations with several studies showing no difference in bleeding risk between the sexes [2–7], while other studies found a higher risk of bleeding in men [8–11]. To our knowledge, there is a lack of large population-based register studies on sex differences in severe bleeding risks in warfarin-treated patients. Therefore, we performed a study using national health registers with the aim to investigate risk factors for severe bleeding after initiation of warfarin including the influence of sex on the incidence of bleeding events.

## Methods

### Data sources

As data sources in this study, we used Swedish national health registers covering the entire population. Data were linked using the personal identity number (PIN) that uniquely identifies all citizens in Sweden. For information on dispensed prescription on warfarin and co-medication, we used the Swedish Prescribed Drug Register (PDR), held by the National Board of Health and Welfare, with data on all dispensed prescriptions in Sweden since July 2005 [12], including Anatomical Therapeutic Chemical classification (ATC) codes [13]. The coverage of the PDR is high with > 99.7% of all prescriptions being recorded with PINs [14]. Diagnoses corresponding to the indications for warfarin treatment, comorbidity and bleeding diagnoses were identified through the Swedish National Patient Register (NPR) [15–18]. The NPR holds information on primary and up to 30 secondary diagnoses from all hospitalizations, nationwide since 1987 and outpatient encounters since 2001. Diagnoses are recorded by the International Classification of Diseases (ICD) system, and the version used in this study is the 10th version (ICD-10), used since 1997. Additionally, the register holds information on surgical procedures performed at hospitals using the Nordic Classification of Surgical Procedures [19]. Information on cancer, including the date of diagnosis, was retrieved from the Swedish Cancer Register [20]. The Cause of Death Register [21] and the Register of the Total Population [22] hold information on individual’s sex, dates of birth, death and migration. Register data were de-identified for research use.

### Study population and follow-up

**Fig. 1:**
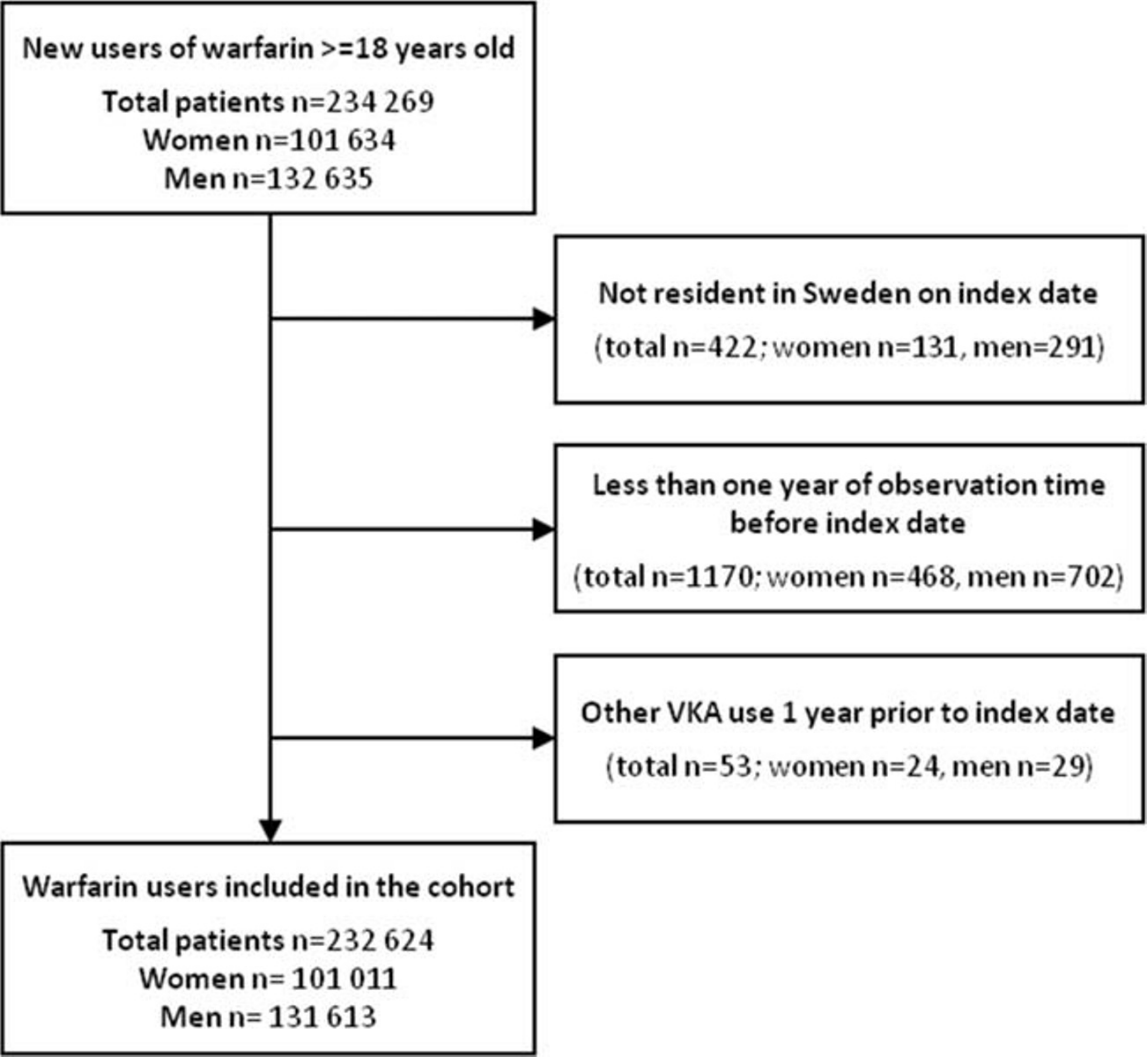
Study flow chart

Women and men over 18 years of age with a dispensed warfarin prescription (ATC code B01AA03) in PDR during the study period January 1, 2007, until December 31, 2011, were included in the study cohort. The inclusion period ended before the introduction of non-vitamin K oral anticoagulants (NOACs). The index date was the first date of a warfarin dispensing during this period. We only included new users, i.e. patients with no vitamin K antagonist (VKA) use 1 year prior to index date. We excluded subjects not resident in Sweden the year before and included the index date (Fig. 1). All patients in the cohort were followed for the occurrence of bleeding events until a maximum of 12 months after the index date, emigration or death, whichever occurred first (intention-to-treat-like approach).

### Indications

The PDR does not hold information on the indication for drug treatment, and therefore, we as proxies included covariates corresponding to the likely indications of warfarin identified in the NPR through the main and secondary discharge diagnosis, as well as the outpatient visit diagnosis (suppl. Table 1). The indications for warfarin included in the analyses were venous thrombosis (VT), pulmonary embolism (PE), venous thromboembolism (VTE) prophylaxis, peripheral systemic embolism, vascular prosthesis, valvular disease, valvular and non-valvular atrial fibrillation (VAF and NVAF), cardioversion, cardiomyopathy, valvular prosthesis and mitral stenosis. For VAF and NVAF, we used diagnoses occurring up to 10 years before the index date, and for the other indications, we used a time window of 3 months before the index date. In the analysis, a patient could be classified as having several possible indications.

### Outcomes

The primary outcome was the first severe bleeding event leading to hospitalization, identified as a main or secondary diagnosis in the NPR. We used the approach for identification of severe bleeding in health registers validated by Friberg et al. [23]. As secondary outcomes, we investigated severe bleeding categorized by anatomical site [23] (Suppl. Table 2).

### Comorbidity and co-medication

In the analyses, we included covariates representing comorbidities and co-medications. For comorbidities, similar definitions and International Classification of Diseases, 10th revision (ICD-10) codes as in two previous studies [14, 24] were used, supplemented with definitions and diagnoses used in the Charlson comorbidity index (CCI) [25, 26] (Suppl. Table 1). Hospital admissions and outpatient contacts for comorbidities were identified up to 10 years before the index date, as were recorded in the cancer registry. Because of the lack of information on international normalized ratio (INR), a modified HAS-BLED score [1, 27] without INR was used for classifying the risk of severe bleeding (Suppl. Table 3). As co-medication, the covariates we included were low-dose aspirin, other antiplatelet agents, NSAIDs, proton pump inhibitors (PPIs), systemic corticosteroids, antidepressants, selective serotonin reuptake inhibitors (SSRIs), antidiabetics and alcohol dependency drugs dispensed within 1 year before index date (Suppl. Table 4). Female hormone therapy was not included due to the different indications and the very different prevalence of use in women and men. However, we performed a restricted analysis excluding patients treated with female hormones. Drugs assessed as having clinically relevant drug interactions with warfarin, i.e. azole antibiotics, macrolides, quinolones, lipid-lowering agents, amiodarone and fluorouracil [28], were also included in the analyses (Suppl. Table 4). These consist of drugs where it is either recommended to avoid concomitant treatment with warfarin (D-interactions) or recommended to monitor INR for warfarin dose adjustment (C-interactions) [29].

### Ethical approval

The study was approved by the Regional Ethical Review Board at Karolinska Institutet (Stockholm, Sweden; Dnr 2013/1850-31/1 and 2014/2215-32).

## Statistics

Descriptive statistics are presented as numbers and proportions. Using multiple Cox regression, we estimated hazard ratios (HR) for severe bleeding in models including as covariates sex, age, comorbidities and co-medication. The HRs were presented with a 95% confidence interval (CI). We finally investigated the effect modification for each covariate by including an interaction term between the covariate and sex in the model. In additional regression models, we adjusted for age as a continuous variable instead of categorical and included co-medications that could lead to drug interactions with warfarin. All analyses were carried out using SAS® software, Version 9.4 (SAS Institute Inc., Cary, NC, USA).

## Results

We included 232,624 patients (101,011 women and 131,613 men) in the cohort. Baseline characteristics of the study population are presented in Table 1. The mean (SD) age was 72.2 years for women and 68.5 years for men, with an excess of persons in the age group ≥ 80 years among females.

**Table 1:** Warfarin study cohort characteristics

	Women		Men	
	*N*	%	N	%
Total	101,011	100.0	131,613	100.0
Age, y mean (SD)	72.2 (13.9)		68.5 (12.8)	
Age group, y				
< 40	4045	4.0	4012	3.0
40–49	3789	3.8	7125	5.4
50–59	6435	6.4	15,695	11.9
60–69	19,165	19.0	37,172	28.2
70–79	33,169	32.8	41,571	31.6
≥ 80	34,408	34.1	26,038	19.8
Indications^a^				
Venous thrombosis	5918	5.9	5751	4.4
Pulmonary embolism	12,734	12.6	12,838	9.8
VTE prophylaxis	1899	1.9	1333	1.0
Peripheral systemic embolism	1144	1.1	938	0.7
Vascular prosthesis	390	0.4	1075	0.8
Valvular disease	5544	5.5	8871	6.7
Valvular atrial fibrillation	4414	4.4	5932	4.5
Non-valvular atrial fibrillation	39,038	38.6	47,470	36.1
Cardioversion	734	0.7	1142	0.9
Cardiomyopathy	790	0.8	2133	1.6
Valve prosthesis	2676	2.6	4816	3.7
Mitral stenosis	245	0.2	95	0.1
Comorbidities/risk factors^a^				
Hypertension^c^	42,475	42.0	46,092	35.0
Diabetes mellitus	10,964	10.9	16,237	12.3
Myocardial infarction	10,080	10.0	19,448	14.8
Ischemic heart disease	17,618	17.4	30,220	23.0
Peripheral vascular disease	3567	3.5	6420	4.9
Congestive heart failure	14,448	14.3	20,348	15.5
Renal failure^c^	2042	2.0	3748	2.8
Liver failure^c^	659	0.7	1132	0.9
Ischemic stroke or TIA^c^	15,355	15.2	18,038	13.7
COPD/emphysema	5482	5.4	5867	4.5
Cancer (excl. Non-melanoma skin cancer)	8644	8.6	11,784	9.0
Alcohol dependency diagnosis^c^	745	0.7	3024	2.3
Platelet or coagulation disorder	1053	1.0	1012	0.8
Prior bleeding^c^	3913	3.9	4941	3.8
Co-medication^b^				
Low-dose aspirin^c^	42,664	42.2	59,273	45.0
Other antiplatelet agents^c^	6861	6.8	11,235	8.5
NSAIDs^c^	22,645	22.4	26,197	19.9
PPIs	28,836	28.5	27,979	21.3
Antidepressants	16,301	16.1	11,133	8.5
Systemic corticosteroids	15,499	15.3	14,032	10.7
Female hormone therapy and contraceptives	22,085	21.9	97	0.1
Alcohol dependency drugs^c^	144	0.1	653	0.5
Antidiabetics	11,151	11.0	17,920	13.6
Interactions^b^				
D interactions	2935	2.9	2701	2.1
Fluconazole	2031	2.0	879	0.7
Sulfamethoxazole	1012	1.0	1947	1.5
C interactions	42,606	42.2	53,605	40.7
HAS-BLED risk score, mean (SD)	2.0 (1.2)		1.8 (1.2)	
HAS-BLED risk score				
Low risk (0–1 points)	36,362	36.0	55,836	42.4
Intermediate risk (2 points)	30,318	30.0	37,937	28.8
High risk (≥ 3 points)	34,331	34.0	37,840	28.8

^a^See supplementary Table 1 for definitions

^b^See supplementary Table 4 for definitions

^c^Included in the modified HAS-BLED score

The most common indications for warfarin treatment were atrial fibrillation, venous thromboembolism (VT and PE), followed by valvular diseases. The indications for warfarin treatment differed between women and men, with VT, PE and NVAF being more common in women compared to men. On the other hand, less women than men had valvular disease. The most common comorbidities were cardiovascular diseases, i.e. hypertension, ischemic heart disease, and congestive heart failure, followed by ischemic stroke or TIA. The frequency of comorbidities also differed between the sexes (Table 1) with, e.g. more women with hypertension but more men with diabetes mellitus, myocardial infarction and ischemic heart disease. Women more often had “high” HAS-BLED risk scores. There were also sex differences in co-medication, with more women treated with NSAIDs, PPIs, antidepressants, SSRIs and systemic corticosteroids, compared to men. More men than women were treated with low-dose aspirin, other antiplatelet agents, alcohol dependency drugs and antidiabetics.

The crude incidence rate of severe bleeding was 37 per 1000 person-years, in women 35 and in men 38 per 1000 person-years. In the analyses of overall risk of severe bleeding, we found a significantly lower risk in women compared to men with a crude HR (95% CI) of 0.94 (0.90–0.98) that was further reduced 0.84 (0.80–0.88) after adjustment for age, indications, comorbidities and co-medication (Table 2).

**Table 2:** Hazard ratios (HR) for severe bleeding: associations with sex, age groups, indications, comorbidities, risk factors for bleeding and co-medication. Cox regression, crude and adjusted for all covariates

	Crude	Adjusted
	HR (95% CI)	HR (95% CI)
Women vs. men	0.94 (0.90–0.98)	0.85 (0.81–0.89)
Age group, y		
< 40	1 (reference)	1 (reference)
40–49	1.23 (0.97–1.55)	1.13 (0.89–1.42)
50–59	1.66 (1.35–2.04)	1.41 (1.15–1.74)
60–69	2.06 (1.70–2.50)	1.67 (1.38–2.03)
70–79	2.80 (2.32–3.38)	2.19 (1.81–2.66)
≥ 80	3.80 (3.15–4.59)	2.88 (2.37–3.50)
Indications^a^		
Venous thrombosis	1.33 (1.22–1.46)	1.32 (1.20–1.44)
Pulmonary embolism	1.12 (1.04–1.19)	1.14 (1.07–1.23)
VTE prophylaxis	1.11 (0.93–1.32)	1.05 (0.88–1.25)
Peripheral systemic embolism	1.81 (1.52–2.16)	1.40 (1.17–1.67)
Valvular disease	1.60 (1.48–1.72)	1.53 (1.35–1.74)
Valvular atrial fibrillation	1.64 (1.50–1.79)	0.96 (0.85–1.07)
Non-valvular atrial fibrillation	1.18 (1.13–1.24)	1.03 (0.98–1.08)
Cardioversion	0.90 (0.70–1.16)	0.86 (0.67–1.12)
Cardiomyopathy	1.02 (0.84–1.23)	0.94 (0.78–1.15)
Valve prosthesis	1.42 (1.27–1.57)	0.90 (0.78–1.04)
Mitral stenosis	2.12 (1.42–3.16)	1.33 (0.88–2.00)
Comorbidities/risk factors^a^		
Hypertension^b^	1.73 (1.66–1.81)	1.22 (1.16–1.28)
Diabetes mellitus	1.68 (1.59–1.78)	1.16 (1.04–1.28)
Myocardial infarction	1.66 (1.57–1.75)	1.07 (0.99–1.17)
Ischemic heart disease	1.63 (1.55–1.71)	1.01 (0.93–1.09)
Peripheral vascular disease	1.93 (1.78–2.09)	1.28 (1.17–1.39)
Congestive heart failure	1.75 (1.66–1.84)	1.19 (1.12–1.26)
Renal failure^b^	3.13 (2.87–3.41)	1.82 (1.66–2.00)
Liver failure^b^	2.07 (1.73–2.46)	1.43 (1.19–1.72)
Ischemic stroke or TIA^b^	1.40 (1.33–1.48)	1.07 (1.00–1.13)
Chronic obstructive pulmonary disease/emphysema	1.79 (1.66–1.94)	1.18 (1.09–1.29)
Cancer (excl. non-melanoma skin cancer)	1.68 (1.57–1.79)	1.33 (1.24–1.42)
Alcohol dependency diagnosis^b^	2.04 (1.80–2.31)	1.79 (1.57–2.05)
Platelet or coagulation disorder	1.43 (1.18–1.74)	1.13 (0.92–1.37)
Prior bleeding^b^	2.63 (2.48–2.79)	1.85 (1.74–1.97)
Co-medication^a^		
Low-dose aspirin^b^	1.34 (1.28–1.39)	0.95 (0.91–1.00)
Other antiplatelet agents^b^	1.60 (1.49–1.71)	1.17 (1.09–1.26)
NSAIDs^b^	1.01 (0.96–1.06)	1.06 (1.00–1.12)
PPIs	1.46 (1.40–1.53)	1.09 (1.04–1.15)
Antidepressants	1.43 (1.34–1.51)	1.23 (1.16–1.31)
Systemic corticosteroids	1.47 (1.39–1.56)	1.20 (1.13–1.27)
Alcohol dependency drugs^b^	1.81 (1.37–2.39)	1.49 (1.11–1.99)
Antidiabetics	1.47 (1.39–1.56)	1.06 (0.96–1.17)

^a^Yes vs no

^b^Included in the modified HAS-BLED score

Table 2 shows the association of covariates with severe bleeding risk. Indications for warfarin associated with a higher risk of severe bleeding were valvular disease with a HR of 1.53 (1.35–1.74), venous thrombosis 1.32 (1.20–1.44), peripheral systemic embolism 1.40 (1.17–1.67) and pulmonary embolism 1.14 (1.07–1.23). Comorbidities associated with the highest risk of severe bleeding were prior bleeding 1.85 (1.74–1.97), renal failure 1.82 (1.66–2.00) and alcohol dependency diagnosis 1.79 (1.57–2.05). Co-medications associated with a higher risk of severe bleeding were “other antiplatelet agents”, NSAIDs, PPIs, antidepressants, SSRIs, systemic corticosteroids and alcohol dependency drugs with HR (95% CI) 1.49 (1.11–1.99).

Among drugs with a known interaction with warfarin, only sulfamethoxazole, ciprofloxacin and simvastatin significantly influenced bleeding risk in an adjusted analysis (data not shown). Including these covariates in the analysis did not change our estimates.

In the analysis of site-specific severe bleedings (Suppl Table 5), women had a lower adjusted risk of CNS bleeding and urogenital bleeding than men, while there was no difference in the risk of GI bleeding and other bleedings.

In the analyses of effect modification (Suppl Table 6), women in the age groups 40–49 and 50–59 had a higher risk of severe bleeding than men. The lower severe bleeding risk in women was independent of indications, HAS-BLED score and comorbidities except renal failure, COPD and prior bleeding. For patients with renal failure, the risk in women exceeded the risk in men. For co-medications, only low-dose aspirin differed from the general pattern with an even more pronounced lower risk of severe bleeding in women (Suppl Table 6).

Adjustments for age as a continuous variable did not change the overall estimates. Neither did the exclusion of patients receiving female hormone therapy and contraceptives lead to important changes in HRs.

## Discussion

In our study, we found that the risk of severe bleeding was significantly associated with the majority of the risk factors included in the HAS-BLED score: age, hypertension, renal and liver failure, ischemic stroke or TIA, prior bleeding, alcohol dependency and co-medication with antiplatelet agents and NSAIDs. We additionally found a higher bleeding risk associated with other factors: diabetes, peripheral vascular disease, congestive heart failure, COPD and cancer. Furthermore, we found an overall lower risk of severe bleeding during warfarin treatment in women compared to men, even more pronounced after adjustment for other factors.

The HAS-BLED risk score has been compared to other risk scores which include additional or other risk factors, such as diabetes and cancer [30–32]. Cancer patients with venous thrombosis are more likely to develop major bleeding during anticoagulant treatment than those without malignancy [33]. Diabetes and congestive heart failure have not previously been associated with a higher bleeding risk during treatment with anticoagulants [34, 35]. A higher bleeding risk during warfarin treatment after 2 years was seen in patients with peripheral artery disease (PAD) [36]. An association between a higher risk of GI-bleeding in patients with COPD has also previously been found [37, 38].

The finding of a lower risk of severe bleeding during warfarin treatment in women is in line with two other studies. A study with elderly patients with AF or VT on VKA treatment with a higher rate of bleeding events in men [10] and a Swedish cohort study on warfarin-naïvepatients showing male sex as an independent risk factor of severe bleeding [9]. The lower risk of CNS bleeding in women found in our study was also in line with another Swedish study on warfarin-treated AF patients [8]. Furthermore, despite the on average lower risk and consistency across analyses stratified on most risk factors, our results showed that in the age groups of 40–49 and 50–59 and in patients with renal failure, women may have a higher risk of severe bleeding than men.

In a study on older patients with VKAs [39], frequent use of NSAIDs or selective COX-2-inhibitors was a strong risk factor for upper gastrointestinal haemorrhage. In our study, however, co-medication with NSAIDs only slightly increased bleeding risk (Table 2). This could be due to the physicians selecting low-risk patient for combination therapy. Concomitant use of aspirin or other antiplatelet drugs in patients with anticoagulants is a known risk factor for bleeding complications [40, 41] with an especially high risk in elderly patients [42, 43]. The risk of low-dose aspirin disappeared in the adjusted analysis (Table 2), which may be explained by a similar selection effect or correlation of aspirin use with other strong risk factors.

Our findings of a lower risk of severe bleeding in women compared to men should be viewed in the light of the risk benefit balance for stroke prevention in women with AF on warfarin, where the differences in the epidemiology of stroke among women and men must be acknowledged. In Sweden, men are more frequently prescribed antithrombotic treatment compared to women [44], and the national US registry data show that women were significantly less likely to use any oral anticoagulant for AF overall and at all levels of CHA_2_DS_2_-VASc score compared to men [45]. Data from a global register study on patients with newly diagnosed NVAF show that the use of anticoagulant therapy for stroke prevention is similar for women and men (approximately 60%), with underuse of anticoagulation therapy in high-risk patients reported for both sexes. At the same time, an overuse of anticoagulation was also reported in individuals with a low risk of stroke [46]. Meta-analyses on sex differences in stroke in AF patients found higher risk of stroke in women [6, 47], and in patients with ischemic stroke and ICH, there were fewer women with good post-stroke functioning compared to men [48], and a possible higher net clinical benefit of VKA treatment in women was suggested in a study showing a slightly higher rate of stroke in women [7]. Our results could partially reflect the fact that the physicians avoid anticoagulation treatment in women with a high bleeding risk to a higher extent than in high-risk men, especially in the older age groups.

### Strengths and limitations

The use of data from population-based healthcare registers with full coverage implies that we avoided recall bias and that there is no selection bias related to the study population. By using the validated bleeding diagnoses by Friberg et al. [23], we ensured the correct identification of the outcome.

With the introduction of the NOACs, prescription patterns changed, and switches between the different antithrombotic substances became common [49].We therefore chose a period before the introduction of NOACs to avoid the complexity with several different antithrombotic substances and indications to consider and a possible selection bias related to the choice of therapy (channelling).

The PDR lacks information on indications, and therefore diagnoses from the NPR corresponding to the indication for warfarin treatment were used as a proxy. Receiving a certain diagnosis depends on patient or physician attitudes and care-seeking behaviour, which potentially could lead to sex differences in diagnoses recorded in the registers. Women have more contact with the healthcare system throughout their lifespan [50–52], which gives them an extra opportunity for disease detection and perhaps more diagnoses/comorbidities. We thus cannot exclude that differential misclassification of diagnoses in women and men could have affected our results. Furthermore, some NSAIDs are available over-the-counter (OTC). Therefore, NSAID use is likely underestimated in our analysis which is based on dispensed prescriptions.

Information on dosage and dates of treatment discontinuation were not available. We performed an intention-to-treat analysis, with the assumption that the warfarin treatment was ongoing throughout the 12-month follow-up period. A gender difference in adherence to warfarin treatment could have contributed to the sex difference in severe bleeding. However, a Swedish nationwide observational study showed no difference between women and men for persistence to warfarin treatment in patients prescribed secondary preventive drugs after stroke [53]. Similarly, differential adherence or persistence could lead to biased effects of other risk factors.

Our results may be confounded by patient frailty. Age and several of the chronic comorbidities we include in the analysis are likely to be associated with frailty, but clinical assessments of frailty were not available. It is noted that the association of bleeding risk with age is only moderately attenuated after adjustment, which could be ascribed to confounding by unmeasured frailty.

We did not have access to diagnoses from primary care, and therefore, we do not have complete information on comorbidities. Hypertension was adjusted for in our analysis, but no data on blood pressure control were available. In a study with data from the Swedish Primary Care Cardiovascular Database, fewer women than men reached target blood pressure [54], but among US adults, women had generally higher hypertension control [55]. Sex differences in hypertension control could potentially contribute to differences in the risk of severe bleeding.

Finally, it is a limitation that our study lacked data on INR and time in therapeutic range (TTR). The adjustment for diagnoses representing the indication for treatment may in part control for systematic differences in INR level and monitoring intensity. For example, valvular disease was associated with a higher bleeding risk. In a study investigating adverse outcomes in women and men with AF taking warfarin in the AMADEUS trial, TTR but not female sex was an independent predictor for combined cardiovascular death and stroke/systemic embolism and clinically relevant bleeding events [11]. Studies based on data from the Swedish national quality registry for AF and anticoagulation have shown that there was no significant difference in TTR between women and men [56, 57]. However, these studies did not assess the direction of the INR deviation from the therapeutic range that could result in either an increased risk of bleeding or an increased risk of thromboembolic events. In a study on epidemiology of subtherapeutic anticoagulation in the USA, women treated for venous thromboembolism were particularly likely to experience low INR [58]. Thus, a lower treatment intensity in women could contribute to a lower bleeding risk.

## Conclusion

In this population-based cohort study in patients on warfarin, the majority of risk factors included in the HAS-BLED score could be confirmed to be significantly associated with a higher risk of bleeding. We also identified an association with several other comorbidities, i.e. diabetes, peripheral vascular disease, congestive heart failure, COPD and cancer. Women had a lower overall incidence of severe bleeding even after adjusting for age, comorbidity and co-medication. The apparent effect of sex was, however, relatively small compared with the effects of other risk factors.

Our findings could partially be explained by selection effects and confounding due to the limitations of our data, including unmeasured confounders, notably treatment intensity and patient frailty.

The individualized dosing may be a key factor, and therefore, exploring risk factors including sex differences in severe bleeding in patients on NOACs with standardized dosing becomes highly relevant. Future studies should also investigate factors not present in healthcare registers that may influence treatment choice and intensity of treatment. For VKA, including information on INR is highly relevant.

## Electronic supplementary material

ESM 1 (DOCX 33 kb)
