# Supplementary material for: Risk factors for severe bleeding events during warfarin treatment: the influence of sex, age, comorbidity and co-medication
Source: Eur J Clin Pharmacol. 2020 Mar 28;76(6):867–76. doi: 10.1007/s00228-020-02856-6 (PMC7239828; doi:10.1007/s00228-020-02856-6)
Supplement: Supplementary file 1 — (DOCX 33 kb) [file 228_2020_2856_MOESM1_ESM.docx]

# Risk factors for severe bleeding events during warfarin treatment – the influence of sex, age, comorbidity, and co-medication.

## European Journal of Clinical Pharmacology

## Authors: Diana M. Rydberg, MD, PhD^1,2^, Marie Linder_,_ PhD^3^, Rickard E. Malmström, MD, PhD^1,2^, Morten Andersen, MD, PhD^3,4^

## Author affiliation: ^1^Department of Medicine Solna, Karolinska Institutet, Stockholm, Sweden; ^2^Clinical Pharmacology, Karolinska University Hospital, Stockholm, Sweden; ^3^Centre for Pharmacoepidemiology, Department of Medicine, Karolinska Institutet, Stockholm, Sweden; ^4^Department of Drug Design and Pharmacology, Faculty of Health and Medical Sciences, University of Copenhagen, Denmark

## Corresponding author: Diana Rydberg, MD, PhD, Clinical Pharmacology, Drug Evaluation Unit, L7:03, Karolinska University Hospital Solna, 17176 Stockholm, Sweden, E-mail: [diana.rydberg@ki.se](mailto:diana.rydberg@ki.se)

Suppl. table 1: Definitions of indications and comorbidity by ICD-10-codes and procedure codes

| **Diagnosis of indication** | **ICD-10-codes or procedure codes beginning with** |
| --- | --- |
| Venous thrombosis | I80-I82 |
| Pulmonary embolism | I26 |
| VTE prophylaxis | NFB, NFC, NGB, NGC |
| Peripheral systemic embolism | I74 |
| Vascular prosthesis | FBJ10, FBL30, FCA50, FCA60, FCA70, FCB40, FCC50, FCD50, FCD70, FDH30, FDJ30, FND96 |
| Valvular disease | I05-09, I33-39, Q232 |
| Valvular atrial fibrillation | I48 and valvular disease |
| Non-valvular atrial fibrillation | I48 and no valvular disease |
| Cardioversion | DF026, DF027 |
| Cardiomyopathy | I255, I42-I43, O903 |
| Valve prosthesis | FGE00, FGE10, FGE20, FGE96, FJF00, FJF10, FJF12, FJF20, FJF96, FKD00, FKD10, FKD20, FKD96, FMD00, FMD10, FMD12, FMD13, FMD96 |
| Mitral stenosis | I342, I050, I052, Q232 |
| **Diagnosis of comorbidity*** |  |
| Hypertension | I10-I15 |
| Diabetes mellitus | E10-E14 |
| Myocardial infarction | I21, I22, I252 |
| Ischemic heart disease | I20-I25 |
| Peripheral vascular disease | I70, I71, I731, I738, I739, I771, I790, I792, K551, K558, K559, Z958, Z959 |
| Congestive heart failure | I099, I110, I130, I132, I255, I420, I425–I429, I43, I50, P290 |
| Renal failure | I120, I131, N032–N037, N052–N057, N18, N19, N250, Z490–Z492, Z940, Z992 |
| Liver failure | B18, K700–K703, K709, K713–K715, K717, K73, K74, K760, K762–K764, K768, K769, Z944  I850, I859, I864, I982, K704, K711, K721, K729, K765, K766, K767 |
| Ischemic stroke or TIA | I63, I64, I679, I693, I694, I698, G450, G451, G452, G453, G458, G459 |
| COPD/emphysema | I278, I279, J40–J47, J60–J67, J684, J701, J703 |
| Cancer (excl. non-melanoma skin cancer) | C00–C26, C30–C34, C37–C41, C43, C45–C58, C60–C76, C77–C80, C81–C86, C88, C90–C97, |
| Alcohol dependency diagnosis | E244, F10, G312, G621, G721, I426, K292, K70, K860, O354, P043, Q860, T51, Y90-91, Z502, Z714 |
| Platelet or coagulation disorder | D65-D69 |
| Prior bleeding | Any severe bleeding diagnoses (suppl table 1) |

*Charlson Comorbidity Index (CCI) ICD 10 code diagnoses from Charlson et al. 1987 and Quan et al.2005 [2,3] with additional diagnoses modified from Forslund et al. EJCP 2014 and Friberg et al. Eur Heart J 2012 [4,5].

Suppl. table 2: Definition of severe bleeding by ICD-10 codes, validated by Friberg et al. [1].

| **Type of bleeding** | **Diagnoses** | **ICD-10 code beginning**  **with** |
| --- | --- | --- |
| CNS bleeding | Subarachnoid haemorrhages | I60 |
|  | Intracerebral haemorrhages | I61 |
|  | Sub- and epidural haemorrhages | I62 |
|  | Traumatic epidural haemorrhages | S064 |
|  | Traumatic subdural haemorrhages | S065 |
|  | Traumatic subarachnoid haemorrhages | S066 |
| GI bleeding | Oesophagal varices with bleeding | I850, I983 |
|  | Gastro-oesophageal laceration-haemorrhage syndrome | K226 |
|  | Bleeding gastric ulcer (subcodes 0,2,4,6 only) | K25 |
|  | Bleeding duodenal ulcer (subcodes 0,2,4,6 only) | K26 |
|  | Bleeding peptic ulcer (subcodes 0,2,4,6 only) | K27 |
|  | Bleeding gastrojejunal ulcer (subcodes 0,2,4,6 only) | K28 |
|  | Acute haemorrhagic gastritis | K290 |
|  | Haemorrhage of anus and rectum | K625 |
|  | Haemoperitoneum | K661 |
|  | Haematemesis, melena and unspecified GI bleeding | K920-K922 |
| Urogenital bleeding | Haematuria | N02 |
|  | Haematuria, unspecified | R319 |
|  | Abnormal uterine and vaginal bleeding | N939 |
|  | Postmenopausal bleeding | N950 |
|  | Haemorrhage in male genital organ | N501A |
| Other bleeding | Conjunctival haemorrhage | H113 |
|  | Choroidal haemorrhage | H313 |
|  | Retinal haemorrhage | H356 |
|  | Vitreous haemorrhage | H431 |
|  | Vitreous haemorrhage in diseases classified elsewhere | H450 |
|  | Ear bleeding | H922 |
|  | Haemopericardium | I312 |
|  | Haemothorax | J942 |
|  | Haemarthrosis | M250 |
|  | Epistaxis | R040 |
|  | Haemorrhage from throat | R041 |
|  | Haemoptysis | R042 |
|  | Pneumorrhagia | R048 |
|  | Haemorrhage from respiratory passages unspecified | R049 |
|  | Haemorrhage not elsewhere classified | R58 |
|  | Haemorrhage and haematoma complicating a procedure not specified elsewhere | T810 |
|  | Iron deficiency anaemia secondary to blood loss (chronic) | D500 |
|  | Anaemia after acute major bleeding | D629 |

Suppl. table 3: Modified HAS-BLED risk score (maximum 8 points)

|  | **Diagnoses and medication** | **ICD-10 code beginning with** | **ATC codes** | **Score** |
| --- | --- | --- | --- | --- |
| **HAS-BLED 1** | Hypertension | I10-I13, I15 | NA | 1 point |
| **HAS-BLED 2** | Renal and/or liver disease | N17-19, K70-77 | NA | 1 or 2 points |
| **HAS-BLED 3** | Stroke or TIA | I63-I64, I679, I693-4, I698, G450-3, G458-9 | NA | 1 point |
| **HAS-BLED 4** | Prior severe bleeding (including anemia) | D50-D53, D55-D64, I60-62, I690-2, S064-6, I850, I983, K250-4, K254-6, K260-2, K264-6, K270-2, K274-6, K280-2, K284-6, K625, K922 | NA | 1 point |
| **HAS BLED 7** | Alcohol related diagnoses, alcohol dependency drugs, and interacting drugs | E244, F10, G312, G621, G721, I426, K292, K70, K860, O354, P043, Q860, T51, Y90-91, Z502, Z714 | N07BB01-05, B01AC, M01A | 1 or 2 points |

**HAS-BLED 6**: age ≥65 years, 1 point

References for the ICD-10 codes in the modified HAS-BLED risk score:

1. Friberg L, Skeppholm M (2016) Usefulness of Health Registers for detection of bleeding events in outcome studies. Thromb Haemost 116 (6):1131-1139. doi:10.1160/TH16-05-0400

2. Charlson ME, Pompei P, Ales KL, MacKenzie CR (1987) A new method of classifying prognostic comorbidity in longitudinal studies: development and validation. Journal of chronic diseases 40 (5):373-383

3. Quan H, Sundararajan V, Halfon P, Fong A, Burnand B, Luthi JC, Saunders LD, Beck CA, Feasby TE, Ghali WA (2005) Coding algorithms for defining comorbidities in ICD-9-CM and ICD-10 administrative data. Medical care 43 (11):1130-1139

4. Forslund T, Wettermark B, Wandell P, von Euler M, Hasselstrom J, Hjemdahl P (2014) Risks for stroke and bleeding with warfarin or aspirin treatment in patients with atrial fibrillation at different CHA(2)DS(2)VASc scores: experience from the Stockholm region. Eur J Clin Pharmacol 70 (12):1477-1485. doi:10.1007/s00228-014-1739-1

5. Friberg L, Rosenqvist M, Lip GY (2012) Evaluation of risk stratification schemes for ischaemic stroke and bleeding in 182 678 patients with atrial fibrillation: the Swedish Atrial Fibrillation cohort study. Eur Heart J 33 (12):1500-1510. doi:10.1093/eurheartj/ehr488

Suppl. table 4: Definitions of co-medication and warfarin interacting drugs by ATC-codes

| **Co-medication** | **ATC-codes** |
| --- | --- |
| Low-dose aspirin | B01AC06 |
| Other antiplatelet agents | B01AC, except for low-dose aspirin |
| NSAIDs | M01A |
| PPIs | A02B |
| Antidepressants | N06A |
| SSRIs | N06AB |
| Systemic corticosteroids | H02AB |
| Female hormone therapy and contraceptives | G03A, G03C, G03D, G03F |
| Alcohol dependency drugs | N07BB |
| Antidiabetics | A10A, A10B |
| **Warfarin interacting drugs** |  |
| Fluconazole# | J02AC01 |
| Voriconazole* | J02AC03 |
| Ketoconazole* | J02AB02 |
| Sulfamethoxazole and trimethoprim# | J01EE01 |
| Ciprofloxacin* | J01MA02 |
| Levofloxacin* | J01MA12 |
| Moxifloxacin* | J01MA14 |
| Erythromycin* | J01FA01 |
| Levothyroxine* | H03AA01 |
| Fluorouracil* | L01BC02 |
| Simvastatin* | C10AA01 |
| Fluvastatin* | C10AA04 |
| Rosuvastatin* | C10AA07 |
| Amiodarone* | C01BD01 |
| Dronedarone* | C01BD07 |

^#^D-interaction, *C-interaction

Suppl. table 5. Incidence rate (IR) of severe bleeding (per 1000 person years, PYs) among women and men on warfarin stratified by bleeding site (for the specific bleeding diagnosis included see suppl.table 3). Adjusted hazard ratios based on Cox regression.

|  | Women (N =101 011, PYs=96 169 ) | | Men (N = 131 613, PYs 125 735) | | Women vs. Men |
| --- | --- | --- | --- | --- | --- |
|  | N | IR (95% CI) | N | IR (95% CI) | Adjusted HR (95% CI) |
| Any severe bleeding^a^ | 3406 | 35.4 (34.2-36.6) | 4759 | 37.8 (36.8-38.9) | 0.84 (0.80-0.88) |
| CNS bleeding | 683 | 7.1 (6.6-7.7) | 969 | 7.7 (7.2-8.2) | 0.79 (0.71-0.87) |
| GI bleeding | 1098 | 11.4 (10.8-12.1) | 1303 | 10.4 (9.8-10.9) | 0.98 (0.90-1.06) |
| Urogenital bleeding | 364 | 3.8 (3.4-4.2) | 966 | 7.7 (7.2-8.2) | 0.41 (0.36-0.47) |
| Other bleeding | 1417 | 14.7 (14.0-15.5) | 1731 | 13.8 (13.1-14.4) | 1.03 (0.96-1.11) |

^a^ as bleeding from multiple sites may occur, the numbers of site-specific bleeding events exceed the total number of severe bleeding events

Suppl. table 6. Effect modification. Incidence rate (IR) of severe bleeding (per 1000 person years) for women and men and HR (women vs. men) stratified by age, bleeding risk, comorbidity and co-medication. Cox regression adjusted for all other factors than the stratification variable. Test for interaction between covariate and sex^a^.

|  | Women (N =101 011) | | | Men (N = 131 613) | | | Women vs. Men | Interaction |
| --- | --- | --- | --- | --- | --- | --- | --- | --- |
| Covariate | N | PYs | IR (95% CI) | N | PYs | IR (95% CI) | Adjusted HR (95% CI) | significance* |
| **Age group, y** |  |  |  |  |  |  |  |  |
| <40 | 46 | 3998 | 11.5 (8.4-15.3) | 65 | 3950 | 16.5 (12.7-21.0) | 0.72 (0.49-1.08) | NS |
| 40-49 | 73 | 3720 | 19.6 (15.4-24.7) | 111 | 7008 | 15.8 (13.0-19.1) | 1.27 (0.93-1.74) | p<0.05 |
| 50-59 | 166 | 6261 | 26.5 (22.6-30.9) | 336 | 15332 | 21.9 (19.6-24.4) | 1.23 (1.01-1.49) | p<0.01 |
| 60-69 | 511 | 18595 | 27.5 (25.1-30.0) | 1067 | 36019 | 29.6 (27.9-31.5) | 0.93 (0.83-1.04) | p<0.05 |
| 70-79 | 1164 | 31794 | 36.6 (34.5-38.8) | 1644 | 39661 | 41.5 (39.5-43.5) | 0.89 (0.82-0.96) | p<0.05 |
| ≥80 | 1446 | 31802 | 45.5 (43.2-47.9) | 1536 | 23765 | 64.6 (61.4-67.9) | 0.70 (0.65-0.75) | p<0.01 |
| **HAS-BLED risk score (%)** |  |  |  |  |  |  |  |  |
| Low risk (0-1 points) | 802 | 35272 | 22.7 (21.2-24.4) | 1238 | 54357 | 22.8 (21.5-24.1) | 0.88 (0.80-0.96) | NS |
| Intermediate risk (2 points) | 1019 | 28915 | 35.2 (33.1-37.5) | 1385 | 36229 | 38.2 (36.2-40.3) | 0.84 (0.77-0.92) | NS |
| High risk (≥3 points) | 1585 | 31983 | 49.6 (47.1-52.1) | 2136 | 35150 | 60.8 (58.2-63.4) | 0.82 (0.76-0.88) | NS |
| **Renal failure** |  |  |  |  |  |  |  |  |
| Yes | 212 | 1731 | 122.5 (106.6-140.1) | 337 | 3193 | 105.6 (94.6-117.4) | 1.16 (0.97-1.38) | p<0.01 |
| No | 3194 | 94438 | 33.8 (32.7-35.0) | 4422 | 122543 | 36.1 (35.0-37.2) | 0.82 (0.78-0.86) |  |
| **COPD/emphysema** |  |  |  |  |  |  |  |  |
| Yes | 303 | 4908 | 61.7 (55.0-69.1) | 348 | 5216 | 66.7 (59.9-74.1) | 1.01 (0.86-1.19) | p<0.05 |
| No | 3103 | 91260 | 34.0 (32.8-35.2) | 4411 | 120520 | 36.6 (35.5-37.7) | 0.82 (0.79-0.87) |  |
| **Prior bleeding** |  |  |  |  |  |  |  |  |
| Yes | 548 | 6364 | 86.1 (79.0-93.6) | 785 | 8881 | 88.4 (82.3-94.8) | 0.96 (0.86-1.08) | p<0.05 |
| No | 2858 | 89805 | 31.8 (30.7-33.0) | 3974 | 116854 | 34.0 (33.0-35.1) | 0.82 (0.78-0.86) |  |
| **Low-dose aspirin** |  |  |  |  |  |  |  |  |
| Yes | 1576 | 40293 | 39.1 (37.2-41.1) | 2561 | 56064 | 45.7 (43.9-47.5) | 0.80 (0.75-0.85) | p<0.01 |
| No | 1830 | 55876 | 32.8 (31.3-34.3) | 2198 | 69672 | 31.5 (30.2-32.9) | 0.89 (0.83-0.95) |  |

^a^ interaction between sex and stratification covariates (see Table 2). NS=not statistically significant. For indication covariates there were no significant interactions. For comorbidities and co-medications only interactions with p<0.05 are reported.
